# Supplementary figures and images for: Modulation of prefrontal couplings by prior belief-related responses in ventromedial prefrontal cortex
Source: Front Neurosci. 2023 Nov 15;17:1278096. doi: 10.3389/fnins.2023.1278096 (PMC10684683; doi:10.3389/fnins.2023.1278096)

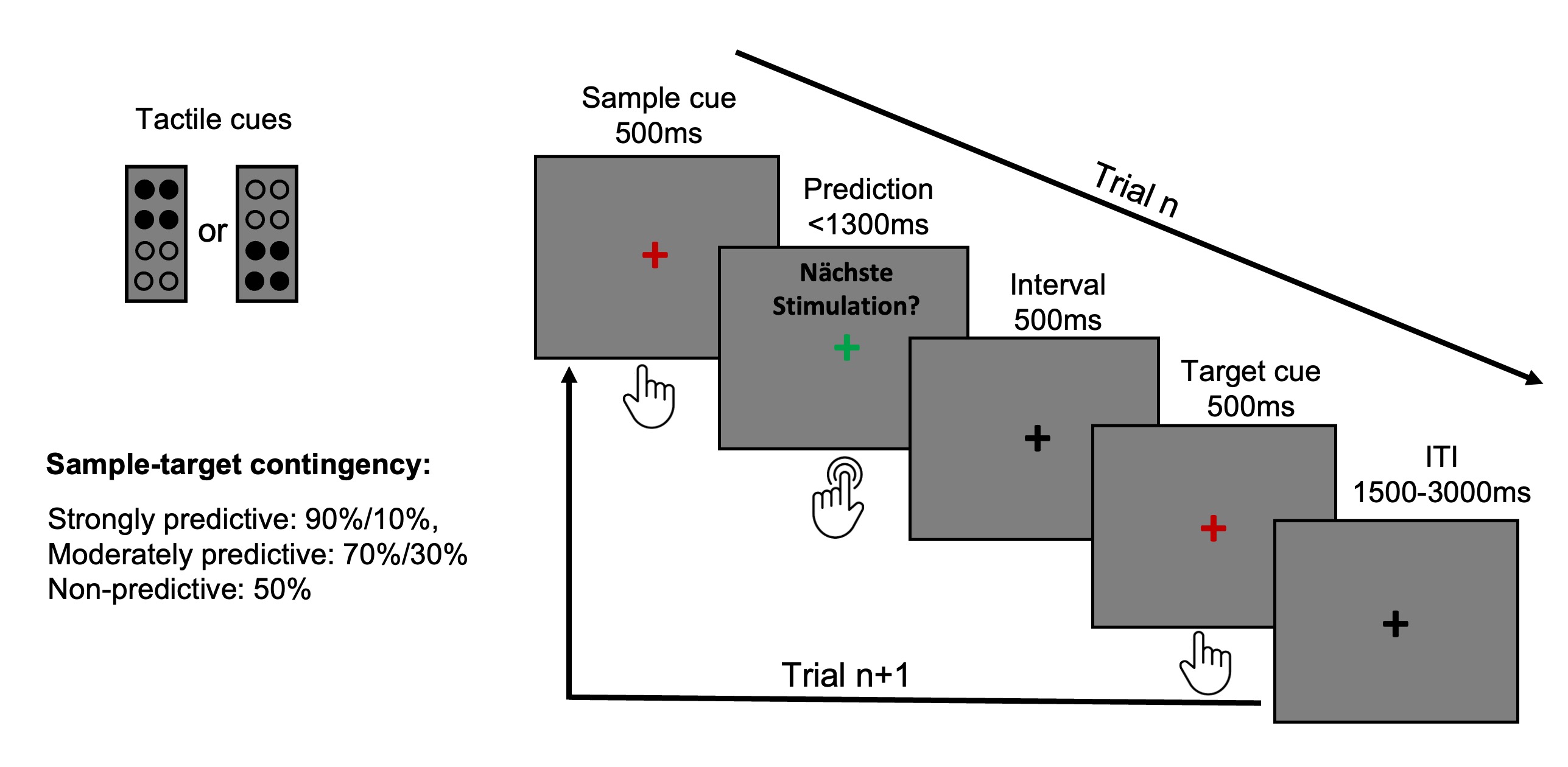

Supplement: Supplementary file 1 [file Image_1.JPEG]

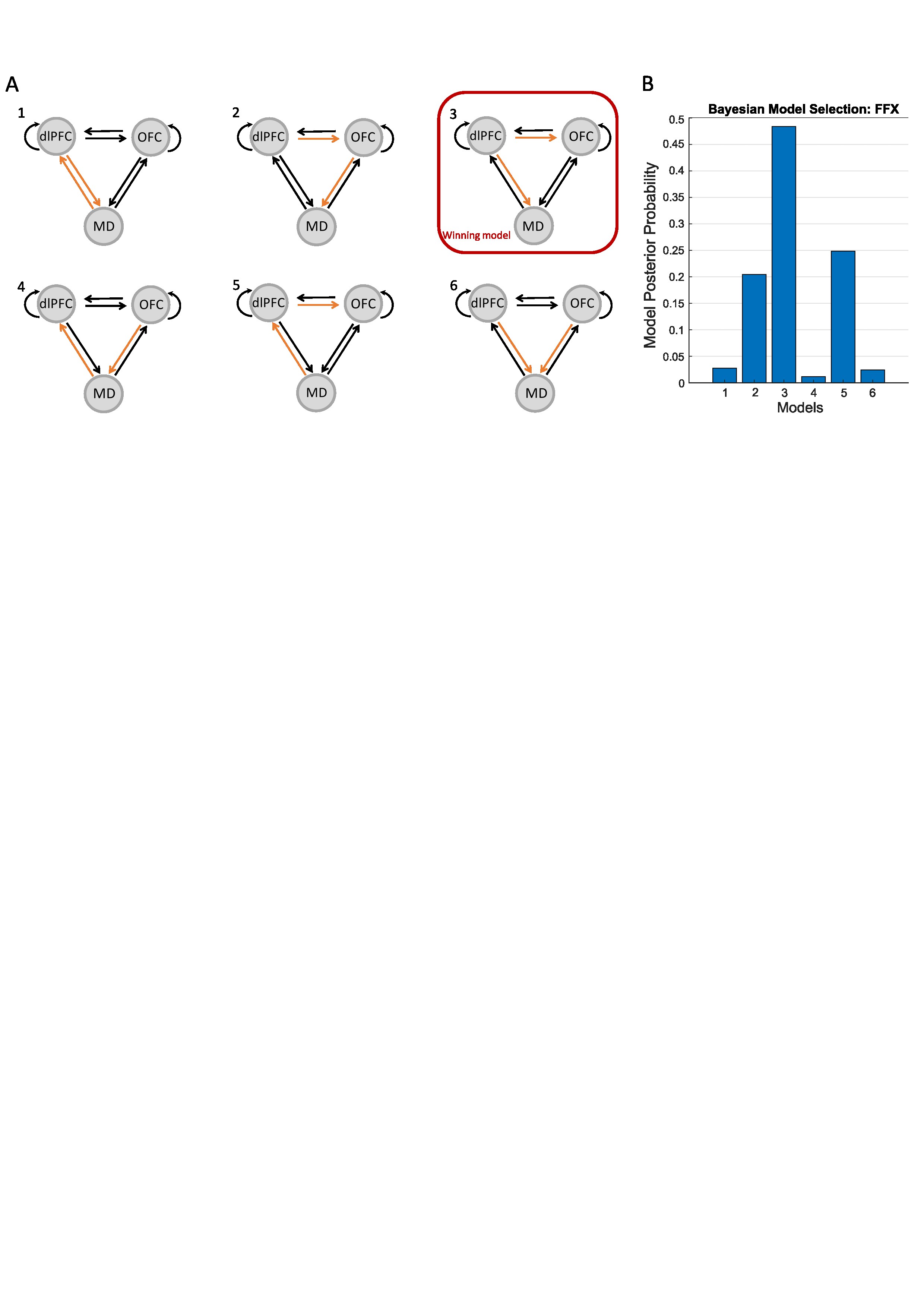

Supplement: Supplementary file 2 [file Image_2.JPEG]
